# Development and internal validation of a diagnostic prediction model for psoriasis severity

**DOI:** 10.1186/s41512-023-00141-5

**Published:** 2023-02-07

**Authors:** Mie Sylow Liljendahl, Nikolai Loft, Alexander Egeberg, Lone Skov, Tri-Long Nguyen

**Affiliations:** 1 Department of Dermatology and Allergy, Herlev and Gentofte Hospital, University of Copenhagen, Gentofte Hospitalsvej 15, 2900 Hellerup, Denmark; 2 Department of Dermatology and Venereology, Bispebjerg and Frederiksberg Hospital, University of Copenhagen, Copenhagen, Denmark; 3 Department of Public Health, Section of Epidemiology, University of Copenhagen, Copenhagen, Denmark

**Keywords:** Diagnosis, Prediction model, Psoriasis, Severity

## Abstract

**Background:**

While administrative health records such as national registries may be useful data sources to study the epidemiology of psoriasis, they do not generally contain information on disease severity.

**Objectives:**

To develop a diagnostic model to distinguish psoriasis severity based on administrative register data.

**Method:**

We conducted a retrospective registry-based cohort study using the Danish Skin Cohort linked with the Danish national registries. We developed a diagnostic model using a gradient boosting machine learning technique to predict moderate-to-severe psoriasis. We performed an internal validation of the model by bootstrapping to account for any optimism.

**Results:**

Among 4016 adult psoriasis patients (55.8% women, mean age 59 years) included in this study, 1212 (30.2%) patients were identified as having moderate-to-severe psoriasis. The diagnostic prediction model yielded a bootstrap-corrected discrimination performance: c-statistic equal to 0.73 [95% CI: 0.71–0.74]. The internal validation by bootstrap correction showed no substantial optimism in the results with a c-statistic of 0.72 [95% CI: 0.70–0.74]. A bootstrap-corrected slope of 1.10 [95% CI: 1.07–1.13] indicated a slight under-fitting.

**Conclusion:**

Based on register data, we developed a gradient boosting diagnostic model returning acceptable prediction of patients with moderate-to-severe psoriasis.

**Supplementary Information:**

The online version contains supplementary material available at 10.1186/s41512-023-00141-5.

## Introduction

Psoriasis is a chronic inflammatory disease affecting primarily the skin with a worldwide prevalence of 2–3% and up to 8% in Denmark [1, 2]. The disease severity ranges from mild forms affecting limited non-sensitive areas to very severe cases with involvement of the entire body surface. The severity of the disease is primarily assessed based on the involvement of skin according to body surface area (BSA, ranging from 0 to 100%), psoriasis area and severity index (PASI, from 0 to 72), or impact on quality of life based on dermatology life quality index (DLQI, from 0 to 30) [3]. The consensus is that patients scoring BSA>10% or PASI>10 or DLQI>10 in one of these measurement tools are labeled as “current severe psoriasis” and are eligible for treatment with systemic therapy [3, 4]. Others advocate that having psoriasis in sensitive areas like the hands or genitals should be considered when defining moderate-to-severe psoriasis [5].

Several different consensus definitions and guidelines of psoriasis severity exist. The terms “mild,” “moderate,” and “severe psoriasis” are commonly used in literature and clinical settings. However, no standard criteria for these terms exist. Standard definitions of mild, moderate, and severe psoriasis are important in both clinical care and clinical research studies [6, 7].

Despite those definitions of psoriasis severity (including BSA, PASI, and DLQI), most administrative registries do not contain information on clinical severity, as neither skin involvement nor involvement of sensitive areas is commonly recorded in administrative health data. As patients with psoriasis are often treated according to the severity of the disease, a common approach to categorize patients into mild versus moderate-to-severe psoriasis is based on recorded treatment, taken as a proxy for clinical severity. While milder cases are often handled with topical therapies or phototherapies, patients with more severe cases often require systemic therapy. Thus, patients currently being treated or with a history of treatment with systemic therapy, e.g., methotrexate or biological therapy, are considered as having moderate-to-severe psoriasis, whereas patients never having received any of these therapies are considered to have mild psoriasis. This poses an issue to estimate the prevalence of patients with moderate-to-severe psoriasis based on clinical characteristics instead of the treatment using large administrative health databases.

To inform clinical and public health research on psoriasis treatment at the country level, it is crucial to estimate the national prevalence and distribution of the disease severity based on clinical characteristics. In the absence of such information, an option is to develop a diagnostic model predicting the severity status, given variables recorded in national administrative health databases.

Administrative health records are an important source of data for studying the epidemiology of diseases with the possibility to include all patients within the population at a given time [8]. In the Nordic countries, national registries with similar data structure and validity can be linked via a personal identification number for, e.g., medical research [9].

In this study, we aimed to develop a diagnostic prediction model to distinguish mild from moderate-to-severe psoriasis based on clinical characteristics and medication use, using a cohort linked to the Danish national registries.

## Method

We developed and assessed the internal validity of a diagnostic prediction model in a population of Danish patients with psoriasis. We reported this study in accordance with the Transparent Reporting of a multivariable prediction model for Individual Prognosis Or Diagnosis (TRIPOD) statement [10]. We were not able to fully adhere to the current TRIPOD statement, as it was made for traditional regression models; a new TRIPOD statement specific to artificial intelligence models is in progress [11].

### Data and study population

In this study, we identified patients from the Danish Skin Cohort with a psoriasis diagnosis. We used cross-sectional data from the Danish Skin Cohort from 2018 which we linked to the Danish national registries [2].

The Danish Skin Cohort is a prospective cohort established with the aim of studying the natural history and disease course of psoriasis in Denmark and has been described in detail elsewhere [2]. In brief, the cohort comprised randomly sampled adults from the Danish general population as well as patients with a diagnosis of psoriasis verified by clinical dermatologists. The Danish Skin Cohort was conducted between 15 May 2018 and 15 July 2018. Those who accepted the invitation were systematically interviewed. The Danish Skin Cohort included among other information on patient-reported measurements of the currently affected BSA, quantitative measures of touch avoidance, and skin and joint pain obtained using a numeric rating scale (NRS) and DLQI [2].

We used the unique personal identification number assigned by the Danish Civil Registration System [12] to all people with permanent residency in Denmark to link data on collected psoriasis medication from the Danish National Prescription Registry [13] and data on hospitalization outcomes from the Danish National Patient Registry [14]. Furthermore, the Danish National Health Service Register was used to identify dermatologist contacts outside the hospital [15]. The Danish national registries are briefly described in Supplementary 1.

### Outcome

The outcome was the severity status of psoriasis, which we defined as a binary variable (mild/moderate-to-severe), based on two self-reported clinical measurements recorded in the Danish Skin Cohort: BSA and DLQI. We defined moderate-to-severe psoriasis if the patient had reported either BSA ≥8 or DLQI ≥7; otherwise, the patients were categorized as mild psoriasis.

### Predictor variables

To predict psoriasis severity, we considered clinical characteristics and medication use recorded in the Danish national registries (i.e., the Danish National Prescription Registry, the Danish National Health Service Register, and the Danish National Patient Registry) as candidate predictors. We considered ten candidate predictors, based on subject knowledge, treatment, clinical guidelines, and existing literature [3, 4, 16]. The predictors used in the model were all selected based on clinical consideration and experience. For the predictors including a time period of 6 months, the choice was made to capture all the patients with these characteristics in the national registries. This choice is based on the clinicians’ experience of either how often a patient is seen by their dermatologist or how often they have prescribed medication. Along with age and sex, we included claimed ≥ 300g of potent or very potent topical corticosteroids in the last 3 months, in-patient hospitalization in the last 3 months specifically due to psoriasis, initiated systemic non-biologic therapy for psoriasis in the last 3 months, initiated biologic therapy for psoriasis in the last 3 months, received phototherapy in the last 6 months, ≥ 4 dermatologist contacts in the last 6 months, switched systemic non-biologic therapy for psoriasis in the last 6 months, and switched biologic therapy for psoriasis in the last 6 months. Each predictor measurement was coded as a binary covariate.

Predictors are described in detail in Supplementary 2.

### Statistical analysis

We constructed a multivariable diagnostic model for psoriasis severity, using a gradient boosting machine model. Gradient boosting is an ensemble model that combines the prediction of multiple individual learners, constructed as binary classification trees, into a single composite model to reduce bias and variance and achieve better predictive performance compared to individual algorithms [17]. The GBM (Generalized Boosted Regression Modeling) algorithm was used to develop the model. The model development included the following hyper-parameters: number of trees, interaction depth, shrinkage, and cross-validation folds. To choose an appropriate value for *k*-fold cross-validation, we explored the effect of different *k* values on the estimate of model performance. A higher number of *k*-fold cross-validation mean training more models, which can be computationally heavy and time consuming. We aimed for the lowest *k* that consistently yielded a comparable prediction performance as compared with higher *k* values. The number of gradient boosting trees was set to 5000, the number of splits to perform on a tree was 5, shrinkage was 0.1, and the number of cross-validation folds to perform was 3.

We assessed the predictive performance of the diagnostic model in terms of calibration and discrimination. Discrimination refers to the ability to distinguish those with outcome from those without outcome; we measured this performance by means of the c-statistic [18]. The 95% confidence interval for the apparent c-statistic was derived by stratified bootstrap (2000 replicates). Calibration refers to the agreement between the predicted probability and the actual probability; we measured this performance by means of a calibration slope, intercept, and observed-to-expected ratio (O/E ratio) [18]. The calibration slope was estimated using a logistic model; the O/E ratio was calculated as follows: observed-to-expected (O/E) ratio = observed prevalence of outcome/expected prevalence of an outcome. In addition, we visually examined the calibration of our model, by grouping the distribution of predicted values by quintiles. Although this method allowed a visual inspection that had a “rougher” granularity than what would have been depicted by a flexible non-parametric local regression curve, this offered more robustness against outliers.

Missing data were handled with multiple imputation using chained equations (MICE) and has been described in detail in Supplementary 1, and trace line plots for the MICE algorithm are shown in Supplementary 3.

To quantify any optimism in the predictive performance, we performed bootstrapping for internal validation across 500 replicates [19]. The intercept of the logistic recalibration model was computed as the calibration-in-the-large.

We performed the analysis as follows: (1) we handle missing data using multiple imputation; (2) we trained the model and performed cross-validation using the imputed data; (3) we performed an internal validation of our analysis by bootstrapping the whole procedure 500 times (each bootstrapped iteration included steps 1 and 2). As such, this final bootstrapping accounted for the uncertainty of all the analytical steps (imputation and model training).

All data processing and analyses were conducted using R statistical software version 4.1.0 (2021-5-18).

## Results

Of the 4016 psoriasis patients included in the study, 1212 (30.2%) were identified as having moderate-to-severe psoriasis based on BSA and DLQI. The median BSA was 3.0, and the median DLQI was 2.0. Baseline characteristics are presented in Table 1. The majority of the patients were female (55.8%), and the mean age was 59 years. Among the study participants, 71.2% had active psoriasis within the last 12 months.

**Table 1:** Baseline characteristics

	Mild psoriasis ***N*** = 2175 (54.2)	Moderate-to-severe psoriasis ***N*** = 1212 (30.2)	Total ***N*** = 4016 (100)
**BSA**, median (IQR)	1 (1; 3)	10 (15; 30)	3 (1; 10)
Missing, *n* (%)	668 (16.5)		
**DLQI**, median (IQR)	0 (0; 0)	3.0 (0; 11)	0 (0; 0)
Missing, *n* (%)	629 (15.7)		
**Age**, mean (SD)	58.5 (14.2)	57.0 (14.8)	58.7 (14.4)
Missing, *n* (%)	0		
**Sex,** *n* (%)			
Female	1178 (54.2)	677 (55.9)	2240 (55.8)
Missing, *n* (%)	0		
**Clinical candidate predictors**, *n* (%)			
Claimed ≥ 300g of potent or very potent topical corticosteroids in the last 3 months	5 (0.2)	28 (2.3)	34 (0.8)
Missing, *n* (%)	0		
Hospitalization in the last 3 months due to psoriasis	< 3		8 (0.2)
Missing, *n* (%)	0		
Initiated systemic non-biologic therapy for psoriasis in the last 3 months	6 (0.3)	7 (0.6)	14 (0.3)
Missing, *n* (%)	0		
Initiated biologic therapy for psoriasis in the last 3 months		< 3	8 (0.2)
Missing, *n* (%)	0		
Received phototherapy in the last 6 months	5 (0.2)	6 (0.5)	14 (0.3)
Missing, *n* (%)	0		
Have had ≥ 4 dermatologist contacts in the last 6 months	107 (4.9)	96 (7.9)	217 (5.4)
Missing, *n* (%)	0		
Switched systemic non-biologic therapy for psoriasis in the last 6 months	6 (0.3)	3 (0.2)	9 (0.2)
Missing, *n* (%)	0		
Switched biologic therapy for psoriasis in the last 6 months	133 (6.1)	55 (4.5)	201 (5.0)
Missing, *n* (%)	0		
**Psoriasis within the last 12 months,** *n* (%)			
No	459 (22.8)	50 (4.2)	571 (14.2)
Missing, *n* (%)	587 (14.6)		

Some of the candidate predictors were very uncommon; only 0.2% of the patients had, respectively, been hospitalized within the last 3 months due to psoriasis, initiated biologic therapy for psoriasis in the last 3 months, or switched systemic non-biologic therapy for psoriasis in the last 6 months.

**Fig. 1:**
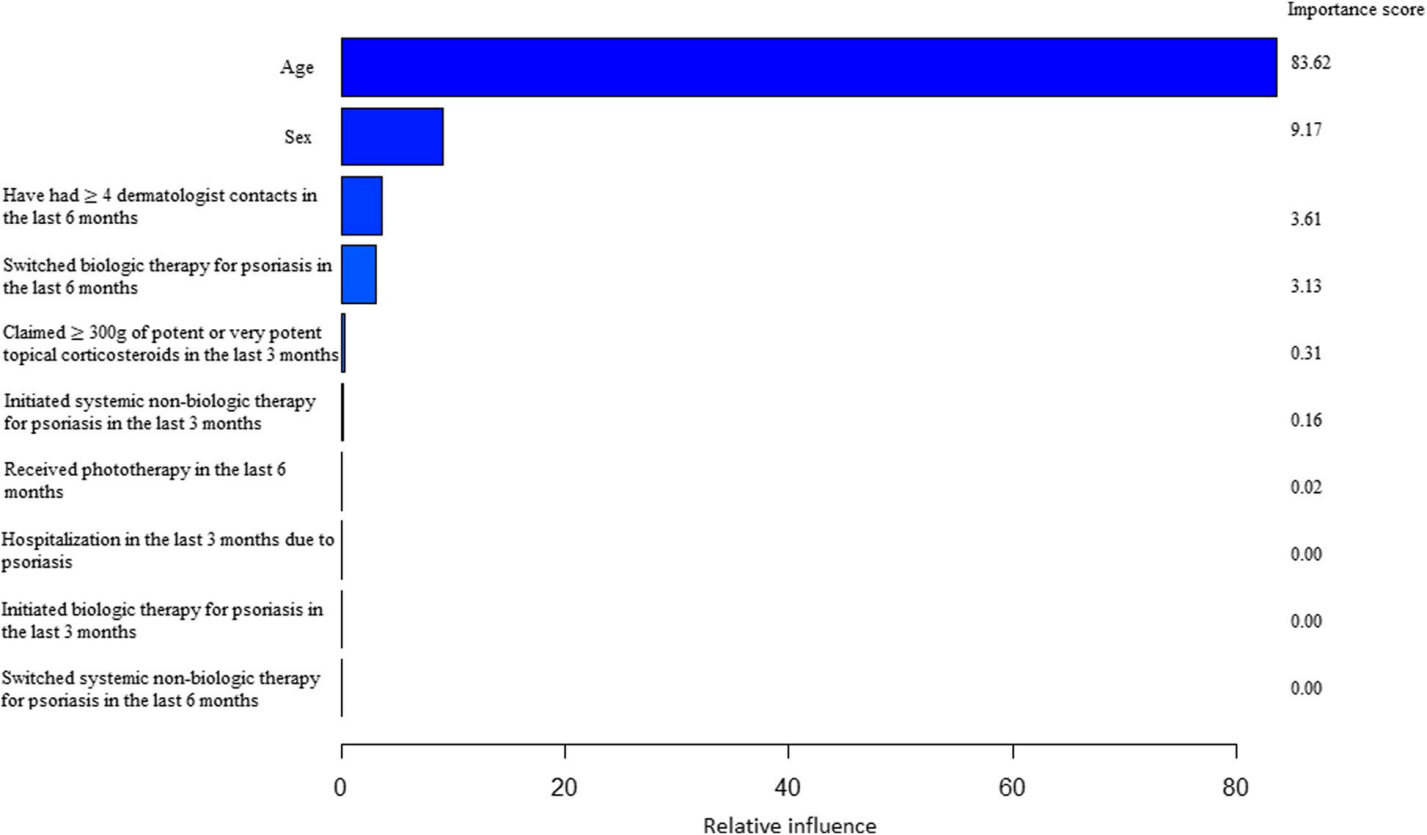
Variable importance of the ten predictors in the gradient boosting machine model. Relative influence (%)

We reported the relative importance of the ten predictors in Fig. 1. The importance measure is not necessarily optimal, since it is mainly driven by the prevalence of the predictors. Although this may be the explanation behind the reported importance scores, we believe the selected predictors reflect the clinical practice routine, as these factors are often used to assess the severity of the disease. The clinical importance of such measurements can also be driven by their prevalence. A factor with a very low prevalence in the population may not offer the clinician much predictive information on average (i.e., over all the patients they see in practice), since only a very few patients would have it expressed. In this sense, reporting the predictor contribution to the model allows one to gain insight into which of these commonly measured variables may actually be clinically important for the diagnosis of psoriasis severity in practice routine. The most important predictors for identifying moderate-to-severe psoriasis patients were age, sex, having ≥ 4 dermatologist contacts in the last 6 months, and switched biologic therapy for psoriasis in the last 6 months.

**Fig. 2:**
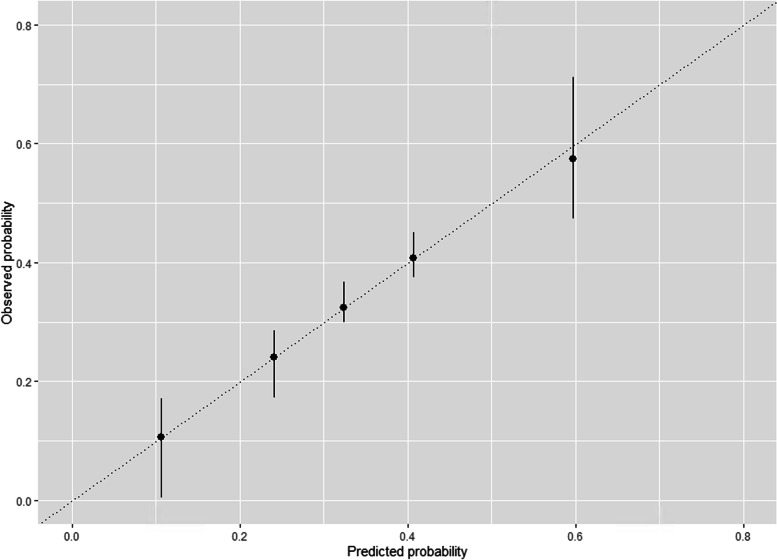
Calibration of the gradient boosting machine model for psoriasis severity by quintiles of predicted probability

After fitting the gradient boosting model to predict the status of psoriasis severity, we reported the calibration by quintiles of our diagnostic model in Fig. 2. In the first quintile, the model underestimated the risk, while it overestimated the risk in higher quintiles. The model yielded an acceptable discrimination with a c-statistic equal to 0.73 [95% CI: 0.71–0.74].

To quantify any optimism in the predictive performance, we performed bootstrapping for internal validation. The stratified bootstrap was used for apparent performance only, to estimate the distribution across 500 replicated samples. The bootstrap-corrected c-statistic was equal to 0.72 [95% CI: 0.70–0.74], the calibration slope to 1.10 [95% CI: 1.07–1.13], and the intercept to 0.06 [95% CI: 0.02–0.09]. The O/E ratio was 0.998, meaning the prevalence of patients with moderate-to-severe psoriasis was likely to be on average approximately equal to 99.8% of the expected prevalence (i.e., mean of predicted probabilities) in the Danish population sharing the characteristics of our cohort. In terms of discrimination, the minimal difference between the apparent and the bootstrap-corrected c-statistic indicated no substantial optimism. As for the calibration, the bootstrap-corrected slope indicated a slight under-fitting.

## Discussion

We have reported a gradient boosting machine model for the estimation of the prevalence of patients with moderate-to-severe psoriasis in the Danish national registries. As with most administrative health care systems, the Danish national registries do not contain clinical information on disease severity. Measures of disease severity were instead captured in a cross-sectional survey [2]. By linking the national claims dataset with the national survey, it was possible to develop a claims-based prediction model for disease severity.

We developed and internally validated a clinical diagnostic prediction model to predict the presence of moderate-to-severe psoriasis in the Danish national registries. Results showed acceptable performance measures and age, sex, having ≥ 4 dermatologist contacts in the last 6 months, and switched biologic therapy for psoriasis in the last 6 months were consistently highly associated with psoriasis severity in the prediction model. The internal validation showed a low optimism in the model based on 500 bootstrap loops. Using this diagnostic tool, it could be possible to estimate the prevalence of patients categorized as moderate-to-severe psoriasis. Thus, this model could possibly estimate the proportion of patients who might be candidates for systemic therapy but not receiving this treatment.

In our study, we found a prevalence of moderate-to-severe psoriasis roughly in line with previous studies. A self-reported community-based population reported 25% of the respondents being in remission (“no symptoms”), 42% of the respondents rated themselves as having mild psoriasis, 24% reported having moderate psoriasis, and 9% rated themselves as having severe psoriasis [20]. A large European survey of patients from clinics revealed a distribution of 9% none, 32% mild, 42% moderate, and 17% having severe psoriasis [21].

Previous studies have primarily used psoriasis therapy based on systemic and biological therapy to estimate moderate-to-severe psoriasis due to a lack of better methods for estimating the severity. Although this approach is in line with the most recent guideline on disease severity proposed by the International Psoriasis Council [6], this does not reflect the disease severity at a specific moment. However, based on clinical experience and the existing literature, the most commonly used clinical tools to assess psoriasis severity are BSA and DLQI—the reason why the definition of psoriasis severity was based on these clinical measurements in this study [3, 4]. The definition of moderate-to-severe psoriasis is based on a proxy for the self-reported BSA and DLQI, which are used in clinical practice routines. The issue of proxy-based definition is relevant to the many definitions of psoriasis severity, but not alone, as it also concerns many medical conditions. However, the outcome definition based on BSA and DLQI measurements reflects the clinical practice routine in Denmark more accurately than a data-driven construction of the outcome. The use of patient-reported BSA has previously been validated and shown to reflect accurately physician-reported BSA scores [22]. Furthermore, a total of 80.4% of psoriasis patients included in the cross-sectional survey reported that their disease had been diagnosed by a physician, predominantly dermatologists [2].

There is no clear-cut definition of mild/moderate/severe psoriasis based on BSA, but patients with BSA≥10 or DLQI≥10 or PASI≥10 are candidates for systemic treatment due to the existing international guidelines. The definition of outcome deviates from “standards” as a safety margin and due to impact on quality of life. Furthermore, a BSA≥10 in the clinical rule “rule of ten” categorizes patients as suffering severe psoriasis. We wanted to include patients with moderate psoriasis in addition to patients with severe psoriasis. As for the dermatology life quality index (DLQI), the effect of the disease on quality of life is categorized as none (DLQI of 0–1), small (DLQI of 2–5), moderate (DLQI of 6–10), very large (DLQI of 11–20), and extremely large (DLQI of 21–30) [23]. In other words, a DLQI ≥7 means psoriasis has a moderate effect on the quality of life of patients. Some of the predictors may systematically imply the disease severity (e.g., hospitalizations and biologic therapy), which makes them relevant to include as potential predictors. Our results showed that those predictors are not necessarily the most informative for the identification of patients with moderate-to-severe psoriasis, because of their very low prevalence. Sex and age were the two predictors with the highest association, followed by patients having ≥ 4 dermatologist contacts in the last 6 months and switched biologic therapy for psoriasis in the last 6 months. This suggests that such predictors might be more informative than the ones classically suggested for the diagnosis of patients with moderate-to-severe psoriasis.

The use of machine learning for prediction in clinical practice and real-world use is complex. To handle multiple layers of complexity—due to, e.g., ensemble models rather than linear equations—the implementation of such tools often requires the development of a specific software system due to the black-box nature. For now, we intend to use our prediction model to estimate the prevalence of moderate-to-severe psoriasis at the national level using registry data in a further study. To mitigate the potential bias in the prevalence estimate of moderate-to-severe psoriasis due to possible misspecification of the prediction model, both the use of inverse probability weighting (IPW) and the use of double robust methods could be considered. Uncertainty could be handled by bootstrapping the data to estimate confidence intervals for the predicted probabilities.

The difficulty of applying such models is largely discussed within the field and with an aim to make the use of these tools more tangible in the future [24, 25].

Our work is to be considered under some limitations. The study limitations included a small number of patients in some of the predictor categories, which might have limited their contribution to the final model. Information of non-responders to the survey was unavailable, and caution is therefore recommended in generalizing our findings (i.e., possible selection bias). Furthermore, we only included adults, and results may therefore not apply to children/adolescents. Some patients had missing data of psoriasis severity which were handled with multiple imputations. The internal validation we performed informs on how our model would likely perform in random samples drawn from the same source population—that is the Danish population of psoriasis patients. Therefore, without further external validation, our model applies only to the Danish population*.* There is no commonly accepted definition of mild and moderate-to-severe psoriasis; hence, this study included two definitions of severity measures of psoriasis. The definition of psoriasis severity was based on BSA and DLQI. Both definitions of psoriasis were based on self-reported outcome measures from the Danish Skin Cohort, yet all patients reporting a history of psoriasis were diagnosed by a physician. The model was based on international guidelines to estimate the proportion of patients who are candidates for systemic treatment. Guidelines are continuously updated on the basis of new knowledge; it will be necessary to update the model to adapt existing guidelines in a future time.

## Conclusion

We have reported a model predicting which patients would have moderate-to-severe psoriasis (as defined by BSA and DLQI) in the Danish National registries. The gradient boosting machine model obtained an acceptable risk prediction for moderate-to-severe psoriasis patients. This model may allow one to identify the group of patients with moderate-to-severe psoriasis in the national registries.

## Supplementary Information

**Additional file 1: Supplementary 1.** Method. **Supplementary 2.** Definition of predictors. **Supplementary 3.** Trace line plots for the MICE algorithm.

## Data Availability

The data that support the findings of this study are available from Statistics Denmark, but restrictions apply to the availability of these data, which were used under license for the current study, and so are not publicly available. Data are however available from the authors upon reasonable request and with permission of Statistics Denmark. Please contact the corresponding author for a data analysis agreement as a collaboration effort.
