# Supplementary material for: Development and internal validation of a diagnostic prediction model for psoriasis severity
Source: Diagn Progn Res. 2023 Feb 7;7:2. doi: 10.1186/s41512-023-00141-5 (PMC9903561; doi:10.1186/s41512-023-00141-5)
Supplement: Supplementary file 1 — Additional file 1: Supplementary 1. Method. Supplementary 2. Definition of predictors. Supplementary 3. Trace line plots for the MICE algorithm. [file 41512_2023_141_MOESM1_ESM.docx]

Supplementary

Supplementary 1: Method

The Danish National Patient Registry contains information on all inpatient and outpatient hospital consultations in Denmark [1]. The Civil Registration System contains information on sex, date of birth and updated information on vital status and emigration [2]. Since 1994 detailed and accurate information on all pharmacy-dispensed medications has been registered in the Danish National Prescription Registry according to the Anatomical Therapeutic Chemical classification [3]. The Danish National Health Service Register contains information of activities of health professionals contracted with the tax-funded public healthcare system [4].

Missing data were handled with multiple imputation using chained equations (MICE). Briefly, each incomplete variable value was imputed a plausible value, based on a separate parametric model conditional on all other variables. Doing so assumed that the missing values could be recovered given the other observed variables (i.e. missing-at-random mechanism assumption). At the time of data collection, missing reports of BSA or DLQI were likely due to the absence of current active psoriasis; these underlying missing values may be predicted from other data as the medication use the last three or six months, given the chronic nature of the disease. (Note, however, this assumption is untestable and residual bias may occur.) The imputation model included all candidate predictor variables and the outcome variable. Instead of imputing directly the predicted hypothetical values returned by the parametric model, we used predictive mean matching, which imputed observed values with a similar predictive mean to avoid bias due to possible misspecification of the parametric model. Values for missing data were imputed 20 times (based on the percentage of incomplete cases) [5], and the maximum number of iterations was set to 50. Estimates obtained across the 20 imputed data sets were then pooled using Rubin’s rules [6].

Supplementary 2: Definition of predictors

| **Predictor** | **Definition** | **ICD10/ATC codes** | **Registry** |
| --- | --- | --- | --- |
| 1 | Claimed ≥ 300g of potent or very potent topical corticosteroids in the last 3 months | ATC codes  Potent: D07AC**, D07BC**, D07CC**, D07XC** D07AX52  Very potent: D07AD**, D07BD**, D07CD**, D07XD** | The Danish National Prescription Registry |
| 2 | Hospitalization in the last 3 months due to psoriasis | ICD10 codes  L400***, L404***, L409*** | The Danish National Patient Registry |
| 3 | Initiated systemic non-biologic therapy for psoriasis in the last 3 months | ATC codes  L04AX03, L04AD01, L04AX07  and medication prescribed at the hospital, OPR codes  ML04AX03, BWHA115, ML04AD01, BOHJ20, D05BB02, ML04AX07, BOHJ28B | The Danish National Prescription Registry and the Danish National Patient Registry |
| 4 | Initiated biologic therapy for psoriasis in the last 3 months | ATC codes  L04AB01, L04AB04, L04AB05, L04AC05, L04AC10, L04AC12, L04AC13, L04AC16  and medication prescribed at the hospital, OPR codes  BOHJ18A2, ML04AB01, BOHJ18A1, ML04AB04, BOHJ18A3, ML04AB05, BOHJ18A5, ML04AC05, BOHJ18B3, ML04AC10, BOHJ18B5, ML04AC12, BOHJ18B6, ML04AC13, ML04AC16, BOHJ18B7 | The Danish National Prescription Registry and the Danish National Patient Registry |
| 5 | Received phototherapy in the last 6 months | ATC code  MD05BA**  OPR codes  BNGA** | The Danish National Prescription Registry and the Danish National Patient Registry |
| 6 | Have had ≥ 4 dermatologist contacts in the last 6 months | Specialty code 1,2 in Dermato-venerology ‘04’ | The Danish National Health Service Register |
| 7 | Switched systemic non-biologic therapy for psoriasis in the last 6 months | ATC and OPR codes as predictor 3 | The Danish National Prescription Registry and the Danish National Patient Registry |
| 8 | Switched biologic therapy for psoriasis in the last 6 months | ATC and OPR codes as predictor 4 | The Danish National Prescription Registry and the Danish National Patient Registry |
|  | Male; Female |  | The Danish Civil Registration System |
|  | Age in years |  | The Danish Civil Registration System |

All predictors including medication use were based World Health Organizations International Classification of Diseases (ICD10) and Anatomical Therapeutic Classification (ATC) system.

Predictor 1 was defined as *‘Claimed ≥ 300g of potent or very potent topical corticosteroids in the last 3 months’* and obtained from Danish National Prescription Registry. The predictor included a minimum of two collections and 300g of doctor-prescribed corticosteroids from a Danish pharmacy during a period of three months from the date of the survey. Topical therapy included topical corticosteroids alone or in combination with topical vitamin D analogues for psoriasis. Potent and very potent topical corticosteroids were defined according to the World Health Organizations Anatomical Therapeutic Classification (ATC) system.

Predictor 2 was defined as *‘Hospitalization in the last 3 months due to psoriasis’.* The definition included in-patient hospitalizations with psoriasis as primary diagnosis during a period of three months from the date of the survey. Information on hospitalization was obtained from the Danish National Patient Registry.

Predictor 3 was defined as ‘*Initiated* systemic *non-biologic therapy for psoriasis in the last 3 months’*. The definition included both non-biologic therapy for psoriasis: Methotrexate, Cyclosporine, Acitretin and Fumaric acid esters from the Danish National Prescription Registry and medication prescribed at the hospital. Initiated systemic non-biologic therapy excluded patients who have used any of the medication before. The patient must initiate the therapy during a period of three months from the date of the survey. All patients who have used any of the medication before were excluded.

Predictor 4 was defined as ‘*Initiated* *biologic therapy for psoriasis in the last 3 months’*. The definition included biologic therapy for psoriasis: Etanercept, Infliximab, Adalimumab, Certolizumab pegol, Ustekinumab, Secukinumab, Brodalumab, Ixekizumab, Guselkumab from the Danish National Prescription Registry and medication prescribed at the hospital. The patient must initiate the therapy during a period of three months from the date of the survey. Patients with any use of the medication before were excluded.

Predictor 5 was defined as *‘Received phototherapy in the last 6 months’.* The definition of the predictor included UV light treatment from the Danish National Patient Registry and from the Danish National Prescription Registry during a period of six months from the date of the survey.

Predictor 6 was defined as *‘Have had ≥ 4 dermatologist contacts in the last 6* *months’.* The definition included dermatologist contacts in The Danish National Health Service Register during a period of six months from the date of the survey.

Predictor 7 was defined as *‘Switched systemic non-biologic therapy for psoriasis in the last 6 months’.* This predictor included patients with more than one systemic non-biologic therapy (Methotrexate, Cyclosporine, Acitretin and Fumaric acid esters) which switched to another during a period of six months from the date of the survey. The predictor included both prescription medication from the Danish National Prescription Registry and medication prescribed at the hospital from the Danish National Patient Registry.

Predictor 8 was defined as *‘Switched biologic therapy for psoriasis in the last 6 months’.* This predictor included patients with more than one biologic therapy (Etanercept, Infliximab, Adalimumab, Certolizumab pegol, Ustekinumab, Secukinumab, Brodalumab, Ixekizumab, Guselkumab) and which switched to another during a period of six months from the date of the survey. The predictor included both prescription medication from the Danish National Prescription Registry and medication prescribed at the hospital from the Danish National Patient Registry.

Lastly, age and sex were included as predictors.

Supplementary 3: Trace line plots for the MICE algorithm


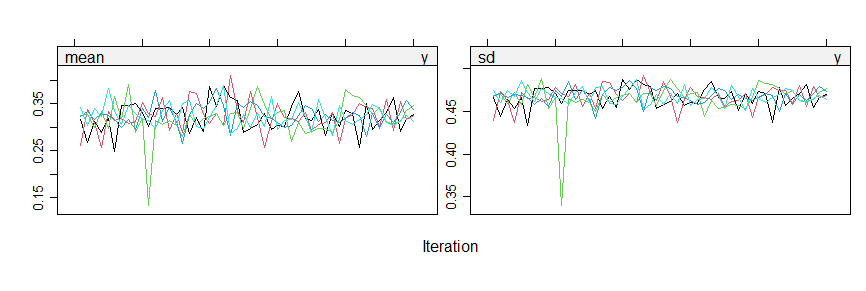


References:

1. Schmidt M, Schmidt SAJ, Sandegaard JL, Ehrenstein V, Pedersen L, Sørensen HT. The Danish National Patient Registry: a review of content, data quality, and research potential. Clin Epidemiol. 2015;7:449–490.
2. Schmidt M, Pedersen L, Sørensen HT. The Danish Civil Registration System as a tool in epidemiology. Eur J Epidemiol. 2014;29:541–549.
3. Pottegård A, Schmidt SAJ, Wallach-Kildemoes H, Sørensen HT, Hallas J, Schmidt M. Data Resource Profile: The Danish National Prescription Registry. Int J Epidemiol. 2017;46.
4. Andersen JS, Olivarius NDF, Krasnik A. The Danish National Health Service Register. Scand J Public Health. 2011;39:34–37.
5. Rubin DB: Multiple Imputation for Nonresponse in Surveys. 1987, New York: John Wiley and Sons.
6. Steyerberg EW, Harrell FE Jr, Borsboom GJ, Eijkemans MJ, Vergouwe Y, Habbema JD. Internal validation of predictive models: efficiency of some procedures for logistic regression analysis. J Clin Epidemiol. 2001;54:774–781.
